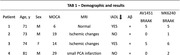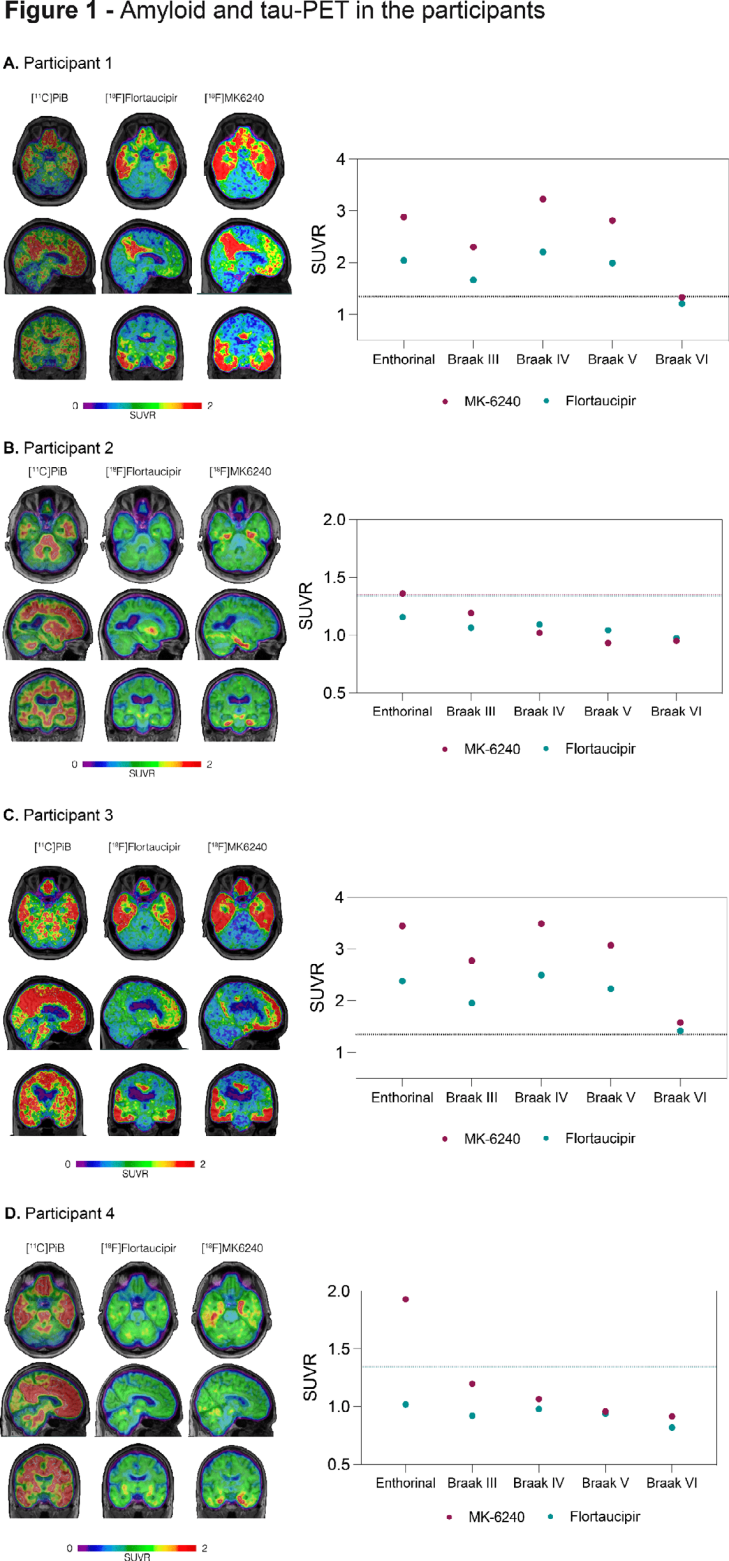# Clinical use of tau PET in Aβ PET positive individuals: a case series

**DOI:** 10.1002/alz.091694

**Published:** 2025-01-09

**Authors:** Vitor Hugo Machado, Guilherme Povala, Bruna Bellaver, Pamela C.L. Ferreira, Livia Amaral, Guilherme Bauer‐Negrini, Firoza Z Lussier, Dana Tudorascu, William J. Jagust, William E Klunk, Val J. Lowe, David N. soleimani‐meigooni, Hwamee Oh, Belen Pascual, Brian A. Gordon, Pedro Rosa‐Neto, Suzanne L. Baker, Tharick A. Pascoal

**Affiliations:** ^1^ University of Pittsburgh, Pittsburgh, PA USA; ^2^ UNIVATES, Lajeado, Rio Grande do Sul Brazil; ^3^ Federal University of Rio Grande do Sul, Porto Alegre, Brazil, Porto Alegre Brazil; ^4^ Helen Wills Neuroscience Institute, University of California, Berkeley, Berkeley, CA USA; ^5^ University of Pittsburgh Alzheimer's Disease Research Center (ADRC), Pittsburgh, PA USA; ^6^ Department of Radiology, Mayo Clinic, Rochester, MN USA; ^7^ Memory and Aging Center, Weill Institute for Neurosciences, University of California, San Francisco, San Francisco, CA USA; ^8^ The State University of New York at Stony Brook, Stony Brook, NY USA; ^9^ Houston Methodist Research Institute, Houston, TX USA; ^10^ Washington University in St. Louis School of Medicine, St. Louis, MO USA; ^11^ Translational Neuroimaging Laboratory, The McGill University Research Centre for Studies in Aging, Montréal, QC Canada; ^12^ Lawrence Berkeley National Laboratory, Berkeley, CA USA

## Abstract

**Background:**

Identifying individuals’ levels of tau PET pathology could prove to be beneficial in clinical settings, given that emerging therapies aimed reducing Aβ seem to be most effective in these individuals. Here, we present the cases of four patients who visited the memory clinic at the University of Pittsburgh Medical Center between June and December 2023 and underwent both Aβ and tau‐PET scans.

**Method:**

These individuals had standard clinical and cognitive outcomes, typical blood tests order in patients with memory impairment, MRI, and, as part of the HEAD study, PET PIB Aβ and two tau PET tracers (MK6240 and Flortaucipir).

**Result:**

All patients presented a primary complaint of progressive memory decline. The results of their blood count, metabolic panel, TSH, and vitamin B12 tests were unremarkable. Patient 1 had a normal MRI, while Patients 2‐4 exhibited mild ischemic changes (Table 1). All patients tested positive for amyloid PET, supporting the diagnosis of AD. However, their tau PET results varied. Patients 1 (MOCA = 6) and 3 (MOCA =19), who were demented with impaired Instrumental Activities of Daily Living (IADL), tested positive for tau PET with Braak stages V‐VI using both tau PET tracers. Conversely, the mild cognitive impairment (MCI) Patients 2 (MOCA = 19) and 5 (MOCA = 29, neuropsych evaluation indicating MCI), who exhibited low tau levels, tested positive for MK6240 but negative for FTP.

**Conclusion:**

All four patients exhibited symptoms and signs consistent with typical amnestic AD presentation, which were confirmed using Aβ PET. Aβ PET presented a similar pattern across all individuals (Figure 1). Tau PET demonstrated greater variability, with lower levels in less cognitively impaired patients. Although tau PET can potentially be useful in identifying individuals in the earliest stages that would benefit from Aβ‐lowering therapies, this small series did not exclude that a low MOCA score combined with Aβ PET could achieve a similar outcome. Larger samples in real‐world clinical contexts are necessary to identify individuals with discrepancies between tau PET uptake and cognitive tests. This would reinforce the need for tau PET markers as complementary tests, singularly capable of identifying individuals across various biological stages to indicate therapies in clinical practice.